# Multienzyme Super-Dosing in Broiler Chicken Diets: The Implications for Gut Morphology, Microbial Profile, Nutrient Digestibility, and Bone Mineralization

**DOI:** 10.3390/ani11010001

**Published:** 2020-12-22

**Authors:** Jacoba Madigan-Stretton, Deirdre Mikkelsen, Elham Assadi Soumeh

**Affiliations:** 1 School of Agriculture and Food Science, Gatton Campus, University of Queensland, Gatton, QLD 4343, Australia; j.madiganstretton@uq.net.au (J.M.-S.); d.mikkelsen@uq.edu.au (D.M.); 2 Bioproton Pty Limited, Acacia Ridge, QLD 4110, Australia

**Keywords:** bone mineralization, broiler chicken, gut morphology, gut microbiota, Natuzyme super-dosing

## Abstract

**Simple Summary:**

Optimizing the gut microbial community and morphometrical traits has become an increasingly prominent area of research due to recent evidence that suggests gut health and functionality affects the production performance of broilers. Creating a diverse microbial population can increase the nutrient digestibility of feed, as the microbes can break down a large portion of macromolecules and convert them into bioavailable substrates to be utilized by the host. A diverse microbiome can be promoted by a variety of additives, including feed enzymes. This study investigated the impact of the application of super-dosing multienzymes on gut morphology, microbial profile, nutrient digestibility, and bone mineralization in broiler chickens. Results found that super-dosing multienzymes improved nutrient digestibility, maintained a diverse microbial population, and tended to increase the overall villi morphology. Bone mineralization was not affected by increasing multienzyme doses. Additionally, the present study found three bacteria that were unique to multienzyme inclusion at a super-dose level.

**Abstract:**

Optimizing gut health has a large impact on nutrient digestibility and bioavailability, and super-dosing feed enzymes may be one solution to achieve this. A 42-day grow-out trial was conducted using 192 Ross 308 broilers to determine if super-dosing Natuzyme at 0 g/t, 350 g/t, 700 g/t, and 1000 g/t dose rates could improve the gut morphology, alter the cecal microbial profile, enhance bone mineralization, and improve nutrient digestibility of a wheat–corn–soybean diet (six replicates per treatment, eight birds per pen). One bird per pen was slaughtered at day 42 and gut morphology, cecal microbial profile, and nutrient digestibility were studied. The addition of enzymes tended to increase the villus height in the duodenum, villus height, width, and crypt depth in the jejunum, and villus width and the number of goblet cells in the ileum. Microbial profiling revealed diverse communities; however, they did not significantly differ between treatment groups. Yet, 700 g/t Natuzyme promoted microbes belonging to the genus Romboutsia and Ruminococcus gauvreauii, while 1000 g/t Natuzyme promoted Barnesiella species. The nutrient digestibility demonstrated a significant improvement in all enzyme doses compared to the control. In conclusion, based on the outcomes of this study, a dose rate of 700 g/t Natuzyme is recommended to improve gut morphology and nutrient digestibility, and promote unique microbes which aid in feed efficiency.

## 1. Introduction

The gastrointestinal tract is composed of an intricate ecosystem of microbiota which plays a fundamental role in nutrient digestion and absorption. The gut microbial profile has a significant impact on overall poultry health, immune response, and growth performance. The resident microbiota is regulated by unique host–microbial and microbial–microbial interactions which have developed through evolution [1,2]. However, the microbiota is strongly influenced by dietary composition, environmental factors, and the health status of the animal [3]. The gastrointestinal tract is the interface between the outside world and the internal body, making it a defensive barrier against harmful pathogens and foreign bodies. Promoting good gut health is fundamental to maintaining a high-performing flock, as healthy birds can devote the majority of their energy uptake to production rather than combating disease. Good gut health results in improved feed conversion ratio, increased weight gain, decreased mortality, and an increased performance index, thus, it is imperative for farmers to promote and maintain good gut health in their flock [3].

Previously, livestock gut health, particularly in poultry, has been modulated by the inclusion of antibiotics in the diets, as they are able to control pathogens through direct manipulation of the gastrointestinal tract microbiota [4]. This manipulation can reduce competition between existing microbes due to a decrease in diversity and abundance, resulting in improved digestion, absorption, and metabolism of essential nutrients [4]. However, over time, animals have become significantly less responsive to antibiotics, and the dose rate has had to increase by 11 times since the initial implementation of antibiotics as growth promotors [5]. The increasing dosage is synonymous with an increase in antimicrobial resistance, which has become a global emerging threat to public health. Therefore, a movement to eliminate antibiotics from livestock production has emerged. Although this is a necessary change, it presents challenges to farmers who are required to seek alternative additives that will promote gut health and production performance.

Recent literature has deemed enzymes to be an advantageous additive for gut health promotion in broilers [6]. From hatching to day 7 of life, the broiler gastrointestinal tract undergoes significant morphological changes, which are heavily influenced by diet. In young birds, the gastrointestinal tract is short, and there is rapid passage rate and limited digestion due to the underdevelopment of villi, and a lack of endogenous digestive enzymes secreted. As the bird approaches 2 weeks of age, the secretion and activity of digestive enzymes increase, which improves nutrient utilization and gut functionality. However, the endogenous and mucosal enzymes have different developmental timetables, which has a direct influence on feed digestibility. Exogenous enzymes are used in animal feed to facilitate the breakdown of antinutritional factors, such as dietary fiber and phytic acid, lower the digesta viscosity, prevent over-fermentation and diarrhea, improve the nutritive value of the feed, and improve production performance and feed efficiency of the animal [7]. Research has demonstrated that the addition of exogenous protease was able to modify the gut morphology, such as increasing villus length and supplementing the lack of endogenous enzyme secretion [8]. In addition, the addition of exogenous protease allows for a reduction in the amount of diet crude protein with no adverse effect on the feed intake, weight gain, and survivability rates in broilers [9].

Super-dosing multienzymes is an emerging practice that is thought to improve nutrient digestibility through synergistic enzyme action. As different enzymes target different compounds, multienzymes should be more effective than single-strain enzymes, as there will be more nutrients acquired from the diet. Poultry naturally produce a plethora of digestive enzymes, however, the digestive process with endogenous enzymes alone still leaves up to 25% of feed undigested due to the presence of antinutritive factors, which the animal cannot combat with endogenous enzymes [7]. There is substantial literature surrounding the effective use of single-strain enzymes, such as phytase, however, publications on multienzyme super-dosing are lacking. Although the literature surrounding this topic is scarce for broilers, Hamdi et al. [10] found that super-dosing phytase improved meat yield and feed conversion ratio (FCR). Furthermore, a review by Cowieson et al. [11] concluded that there may be considerable opportunity to improve production performance by super-dosing phytase, however, the exact mechanism is still unknown. Therefore, it is important to investigate the effects of super-dosing multienzymes to fill the knowledge gap surrounding the effects and mode of action of this nutritional strategy.

It is hypothesized that super-dosing multienzymes from day 0 post-hatch can improve gut morphology and nutrient digestibility due to the increased bioavailable nutrient content, and promote a diverse and stable microbial community, which will ultimately improve flock performance.

## 2. Materials and Methods

All experimental procedures involving the use of animals complied with the “Australian Code of Practice for the Care and Use of Animals for Scientific Purposes” and were approved by the Animal Ethics Committee of the University of Queensland. The animal ethical certificate was obtained prior to the commencement of this trial (number: SAFS/510/18/BIOPROTON).

### 2.1. Birds and Experimental Design

One-hundred and ninety-two 1-day-old mixed-gender broiler chickens (Ross 308) were purchased from a commercial hatchery (Woodlands Hatchery, QLD) and transferred to the isolation shed, a closed shed with isolated cold room panels at the Queensland Animal Science Precinct (QASP) facility at Gatton Campus, University of Queensland. The birds were weighed and randomly assigned to 1 of 4 experimental groups in a randomized complete block design (RCBD). Each experimental diet was fed to 6 replicate pens (1 × 1 m^2^) with 8 birds in each (*n* = 48 per experimental group). The experimental diets (Table 1) were in mash form and included a standard wheat–corn–soybean diet (control) with 4 enzyme inclusion levels (0, 350, 700, and 1000 g/ton). No antibiotics were added as growth promotors. All nutrients were supplied to meet nutrient recommendations (Table 2). The enzyme Natuzyme (Bioproton Pty. Ltd. QLD, Acacia Ridge, Australia) is a multienzyme blend that includes phytase, xylanase, cellulase, amylase, protease, beta-glucanase, and mannanase at specific activities (Table 3), currently recommended at 350 g/t by the manufacturer. The birds had ad libitum access to feed and water for the entire trial period. As per standard procedure, the grow-out period was divided into 3 phases (starter diet: day 1–14; grower diet: day 14–28; finisher diet: day 28–42) and nutrient levels were adjusted accordingly as per Ross 308 guidelines. On day 35, a natural indigestible and inert marker, acid insoluble ash, was added at a level of 0.2% to each experimental diet as a dietary digestibility marker. The lighting program, temperature, and humidity followed the Ross 308 guidelines. The lighting program provided 23 h of light at an 30–40 lux intensity and 1 h dark (less than 0.4 lux) for the first 7 days and a minimum of 4 h of darkness and a light period of 10 lux intensity after 7 days. The temperature was set at 32 °C and 40% relative humidity for the first 7 days and there was a 2 °C reduction per week after 7 days until the temperature reached 24 °C at 27 days and 40% relative humidity. The temperature and relative humidity were maintained until the end of the trial.

At the end of the experiment, one bird with a body weight (BW) similar to the mean BW of the pen was euthanized by cervical dislocation and eviscerated to collect gut tissue for gut morphology, ileal digesta for nutrient digestibility, cecal content for microbial profile, and bone samples for bone mineralization studies.

### 2.2. Gut Morphology

The gastrointestinal tracts from the base of the gizzard down to the rectum were dissected, and sections (~1 cm) were cut from the mid-regions of the duodenum, jejunum, ileum, and cecum, flushed with distilled water, and immersed in 10% formalin solution. Fixed tissues were then loaded into appropriately sized cassettes for further gut histo-morphological analysis. Each fixed intestinal tissue sample was dipped in wax and a 5 mm section was cut and embedded in paraffin (Medite TES Valida embedding station). Embedded intestinal segments were cut at a thickness of 6 μm (Leica semi-automated RM2245 rotary microtome, Leica Microsystems, VIC, Melbourne, Australia) and mounted onto slides. Then slides were stained by hematoxylin and eosin (HE), dried in the oven overnight, and cleaned to be scanned by light microscopy. The slides were scanned by an Aperio ScanScope XT (Leica Microsystems, VIC, Melbourne, Australia) and studied for the villus height, crypt depth, villus width, and the number of goblet cells. Then, the villus surface area was calculated and the villus height to crypt depth ratio measured.

Villus height was measured from the tip of the villus to the crypt between individual villi. Crypt depth was measured from the valley between the bases of the villi to the submucosa. Villus width was calculated from the mean value of villus width at one-third and villus width at two-thirds of the height of the villus. The area between 4 villi was used from 3 cuts per sample to count the number of goblet cells. The average of the 3 measurements was then reported as the number of goblet cells per surface area.

### 2.3. DNA Extraction and 16S rRNA Gene Amplicon Sequencing

Cecal content samples were collected from 1 bird per pen at 42 days of age, post-mortem. Digesta from the cecum was collected into a 10 mL tube. Dissecting instruments were cleaned with 70% ethanol after use on each bird. Samples were put on ice immediately and stored at −20°C prior to DNA extraction.

For each poultry digesta sample, 50 mg was transferred into a sterile 2 mL screw-cap tube containing sterile 0.1 mm and 1.0 mm zirconia beads (total weight 0.4 g; ratio 1:1). Microbial cell lysis was achieved by bead beating in a Qiagen TissueLyser II (Qiagen, Hilden, Germany; 30 Hz, 60 s, 1 repetition conditions), for total genomic DNA (gDNA) extraction. Post lysis, tubes were left for 2 min, for phase separation of the lysate, and each sample supernatant (~600 μL) was then processed for gDNA extraction and purification using the Maxwell 16 blood DNA purification kit (Promega, AS1010, Madison, WI, USA) and the automated Maxwell 16 MDx instrument (Promega, Alexandria NSW, Australia), according to the manufacturer’s instructions. The instrument was set to standard elution volume (SEV) mode and the gDNA eluted into 300 μL of elution buffer. Thereafter, the gDNA samples were quantified and purity verified with the NanoDrop 1000 spectrophotometer (Thermo Scientific, Brisbane, Australia), and subsequently submitted to the Australian Centre for Ecogenomics (ACE; the University of Queensland, Brisbane, Australia) for amplicon sequencing.

The variable region of the 16S rRNA gene was amplified between the V6 and V8 regions, using the universal forward and reverse primer pair of 926F (5′-TCG TCG GCA GCG TCA GAT GTG TAT AAG AGA CAG AAA CTY AAA KGA ATT GRC GG-3′) and 1392wR (5′-GTC TCG TGG GCT CGG AGA TGT GTA TAA GAG ACA GAC GGG CGG TGW GTR C-3′). The gDNA was fragmented and tagged with specific index adapter sequences on both ends of the gDNA fragments using the Nextera XT DNA Library Preparation kit (Illumina, San Diego, CA, USA), enabling dual-indexed sequencing of pooled libraries. The libraries were pooled and sequencing was then carried out on a 2 × 300 bp V3 MiSeq sequencer (Illumina, San Diego, CA, USA). Post-sequencing, quality control, indexing, quantification, and normalization steps were carried out by the sequence provider.

### 2.4. Bioinformatics Analysis

Raw data files were provided by ACE as fasta files. Analysis of these sequencing files were carried out by using Quantitative Insight into Microbial Ecology 2 (QIIME2) [12]. Operational taxonomic units (OTUs) were assigned using the Greengenes 13_8 database [13], using a threshold setting of 97% sequence identity. These data were then normalized to relative abundance using cumulative sum scaling (CSS), a widely used method for normalizing microbial community composition data, and the data were also log2 transformed to account for the non-normal distribution of taxonomic count data, in Calypso Version 8.84 [14]. This software was also used to generate α-diversity parameters, hierarchical clustering and principal coordinate analysis (PCoA) plots. To examine the alpha diversity parameters of each sample, rank tests including richness, evenness, Chao1 index, Shannon index, and Simpson’s index were carried out. The richness examined the number of different OTUs present within each sample and the evenness was a measure of the relative abundance of the different OTUs making up the richness. Additionally, PCoA (beta diversity) was conducted based on Bray–Curtis distance metrics focusing on the top 20 OTUs, to explore how the overall microbiota composition differed across the 24 samples and four diets.

### 2.5. Nutrient Digestibility

Post-processing, the gastrointestinal tract was removed from the carcass. The ileal content was evacuated into a 10 mL O-ring tube and placed on ice and left in a −20 °C freezer until freeze drying occurred. This process was repeated for all 24 birds. The sample was weighed pre-freeze drying and post-freeze drying to calculate dry matter. To determine the ash and organic matter content, 2 g of the sample were placed in a crucible and burned in a muffle furnace for 3 h at 500 °C [15], and it was calculated by the following equation:

Wt% ash = (ashed sample − preashed sample) ∗ 100

Organic matter = 100 − wt% ash

To determine carbon, nitrogen, and sulfur content in the sample, 1 g of sample was placed into a ceramic boat in the Leco CNS 928 combustion analyzer (LECO Australia, NSW, Castle Hill, Australia) and analyzed.

The nutrient ileal digestibility was calculated using the following equation:

Nutrient apparent ileal digestibility (%) = [100 − (Ni × Md)/(Nd × Mi)] × 100

where Ni represents a concentration of the nutrient in ileal digesta; Md represents a dietary concentration of marker; Nd represents a dietary concentration of the nutrient under the study; and Mi represents the concentration of marker in ileal digesta [16].

### 2.6. Bone Mineralization

The meat was removed from the right tibia bone before freezing the bone samples for mineral analyses. The right tibia bone of 24 chickens were defrosted and broken into small pieces using pliers. The entire tibia bone was collected from the right leg of each broiler chicken at 42 days and cleaned of adhering tissue. Bones were dried to a constant weight at 105 °C, then burned to ash in a muffle furnace at 600 °C. Ash was dissolved in concentrated HCl for mineral determination (5 mL of 6 M hydrochloric acid and 35 mL of distilled water), and the solution was filtered into a 250 mL glass bottle and made up to a final volume of 50 mL with distilled water. Thereafter, the Ca and P levels were measured using a Thermo iCAP 6000 series inductive coupled plasma (ICP) spectrophotometer (Thermo Electron Corporation, Str. Rivoltana, 20090 Rodana, Milan, Italy). The Ca and P composition (g/kg in ash) was calculated using iTEVA Analyst software (Cambridge, UK).

### 2.7. Statistical Analysis

Analysis of variance was performed on the least square means (LSM) values of the pens as an experimental unit using mixed models of SAS [17]. All values of *p* ≤ 0.05 were deemed statistically significant, and all values of 0.05 ≤ *p* ≤ 0.1 were considered a tendency. The reported LSM was separated using Tukey’s post hoc test.

## 3. Results

Multienzyme actual activities for different Natuzyme dose rates were very close to the expected activities (Table 4).

### 3.1. Gut Morphology

There were no significant effects in any of the gut morphological parameters when Natuzyme was added into the standard diet in increasing dose rates (Table 5). As the enzyme dose increased, the villus height in the duodenum (*p* = 0.06) and jejunum (*p* = 0.09) tended to increase. The increasing dose rate of Natuzyme also tended to increase crypt depth (*p* = 0.10) and villus width in the jejunum and ileum (*p* = 0.10).

**Figure 1:**
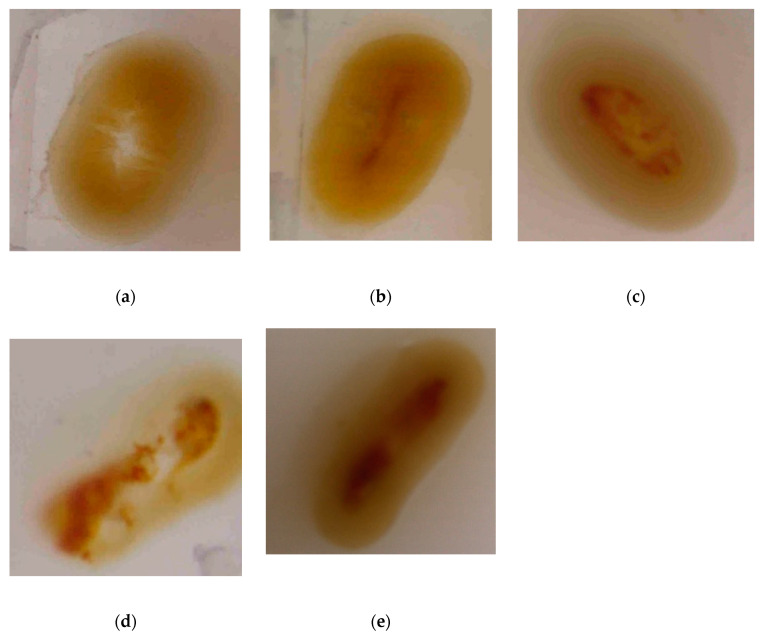
Bloodiness scale: (**a**) grade 1 (no blood present); (**b**) grade 2: thin lining of blood in the center of the gastrointestinal tract; (**c**) grade 3: patchy blood scattered throughout the center of the GIT; (**d**) grade 4: patchy, thick blood in the center of the GIT; (**e**) grade 5: thick, consistent blood throughout the GIT.

Anecdotal data revealed that several gut samples from birds fed with 1000 g/t Natuzyme had blood observable on a macroscopic level (Figure 1).

### 3.2. Microbial Community Diversity

For all samples analyzed, following sequencing quality control, amplicon clustering, and taxonomic classification, a total of 949,364 sequence reads were obtained. Per sample, sequence reads ranged from a minimum of 17,209 to a maximum of 42,441, and clustered into 1103 OTUs. A rarefaction plot was generated indicating the total number of sequence reads and richness obtained per sample (Supplementary Material Figure S1). This allowed for the determination of whether the appropriate sequence depth was achieved per sample, thereby ensuring that the microbial community in each sample was robustly represented. The relative abundance of combined experimental groups is presented in Table 6.

**Figure 2:**
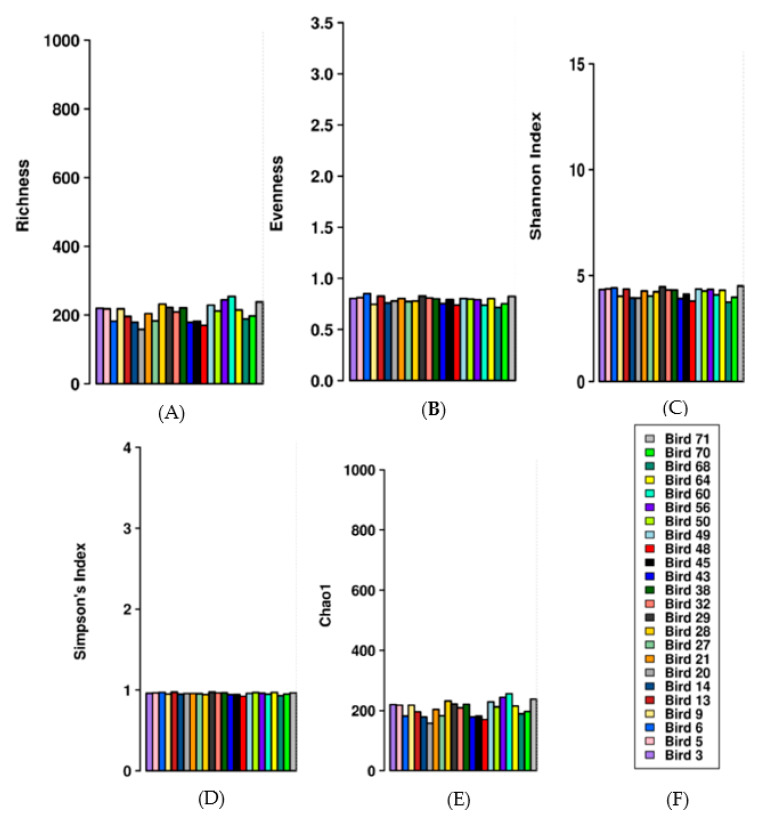
Alpha diversity parameters of caecum microbial profile: richness (**A**), evenness (**B**), Shannon index (**C**), Simpson’s index (**D**), and Chao1 index (**E**) in 24 sampled birds (**F**).

Alpha diversity parameters including richness, evenness, Shannon index, Simpson’s index, and Chao1 index were examined (Figure 2). Richness examined the number of different OTUs present within each sample (Figure 2A), while evenness measured the relative abundance of the different OTUs comprising the richness (Figure 2B). The results suggest that the microbial profiles in the digesta sampled from the poultry birds fed one of four experimental diets shared similar richness and evenness when compared, and these observations were not different (*p* ≥ 0.05). However, the Shannon indices observed in this study for all 24 samples were over 3, indicative of the existence of a highly diverse microbiota per sample (Figure 2C), as Shannon index values above 3 are regarded as indicators of a diverse microbial community, in terms of richness and evenness [18]. Additionally, the Simpson’s indices, which account for both the number of OTUs present and their relative abundance per sample, suggests that all samples had similar OTU diversity (Figure 2D). Chao1 index indicated no difference in OTU abundance between all the samples (Figure 2E). Thus, overall, the alpha diversity parameters indicate that the digesta sampled contained a highly diverse microbiota, which was similar in abundance across all 24 samples.

The rank test indicates the number of different species present in each sample and the evenness rank test measures the relative abundance of the different species making up the richness, while the Shannon index accounts for both abundance and evenness of species present in each sample.

**Figure 3:**
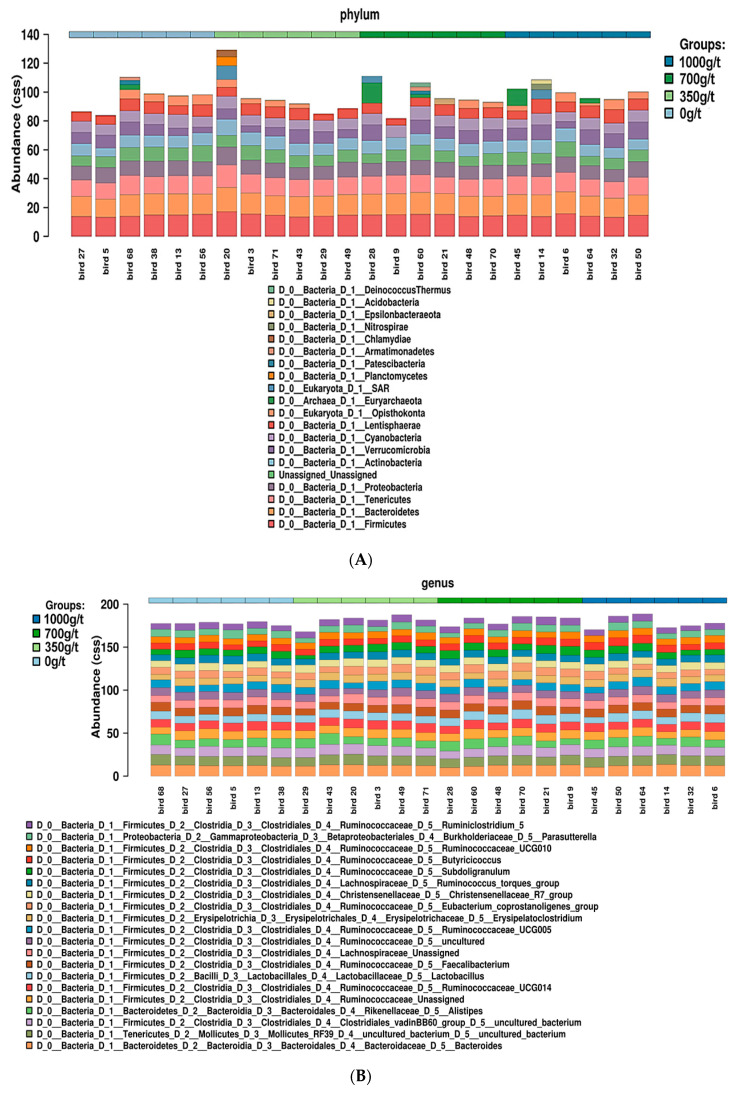
(**A**) Relative abundance of operational taxonomic units (OTUs) present in each poultry digesta sample, at a phylum level. (**B**) Relative abundance of OTUs present in each poultry digesta sample, at a genus level.

**Figure 4:**
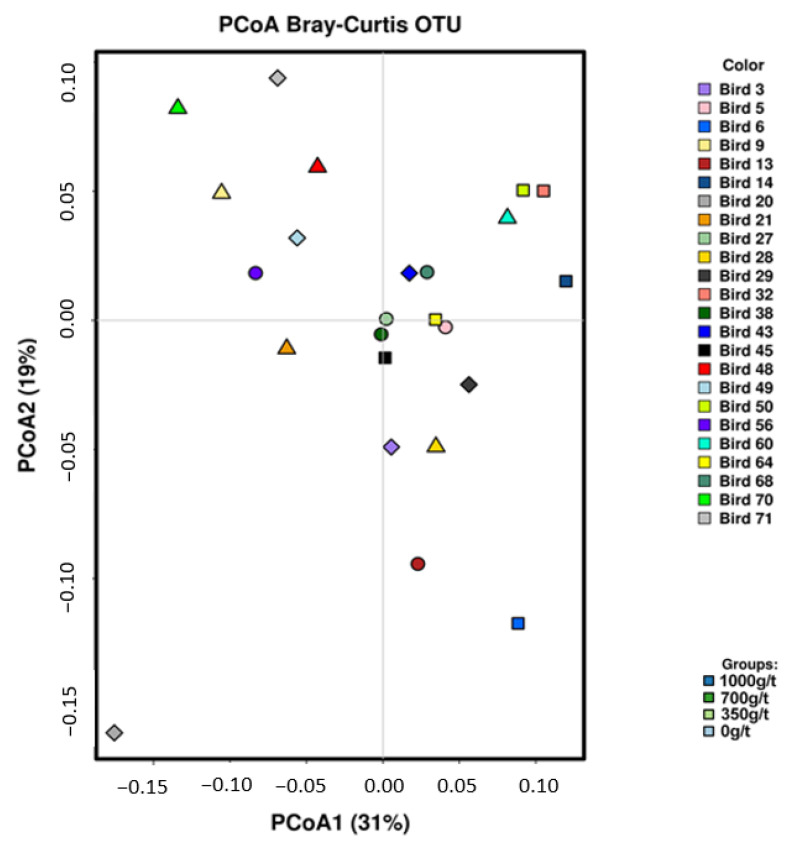
Principal coordinate analysis (PCoA) of microbiota based on Bray–Curtis distance for OTUs.

Analysis of the 20 most abundant phyla and genera present across all 24 samples (Figure 3A,B, respectively) indicates that no differences in the microbial diversity in terms of relative OTU abundance were observed between birds on the four treatment diets. No distinct grouping of microbial communities was revealed, with PCoA1 and PCoA2 covering 31% and 19% of the total microbiota, respectively (Figure 4).

**Figure 5:**
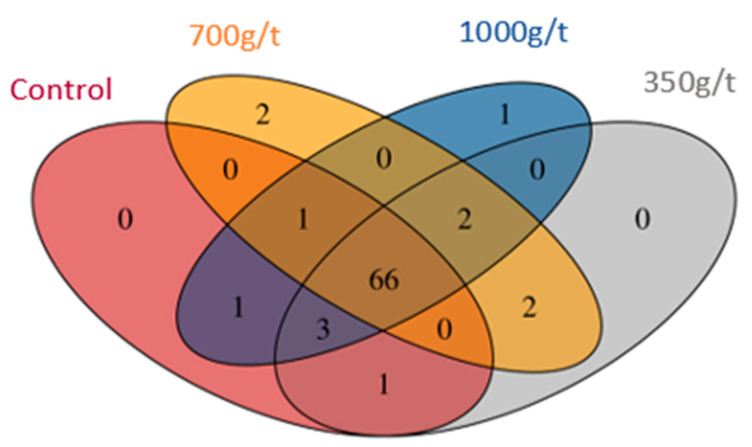
Core microbiota analysis was carried out at the genus level, including the top 100 relatively abundant OTUs present, to visualize the differences between the samples, based on diet groupings.

However, core microbiota analysis of the top 100 abundant genera revealed that 66 OTUs were core to all four diet treatments, yet only three appeared to be uniquely associated with 700 g/t Natuzyme (two unique OTUs) and 1000 g/t Natuzyme (one OTU) (Figure 5). Specifically, the OTUs related to the genus Barnesiella were identified to be unique to 1000 g/t Natuzyme, while the genus Romboutsia and the microorganism Ruminococcus gauvreauii were identified to be unique to 700 g/t Natuzyme.

### 3.3. Nutrient Digestibility

The effects of Natuzyme inclusion at different dose rates on nutrients’ apparent ileal digestibility were highly significant (Table 7). As the dose rate increased, the nitrogen/crude protein and organic matter digestibility both increased (*p* < 0.001). In terms of nitrogen, 700 and 1000 g/t Natuzyme revealed a significantly higher digestibility than the control (no enzyme) and 350 g/t, however, there is a general linear increase in digestibility as enzyme dose rate increases. In terms of organic matter, the experimental diets with Natuzyme included at all dose rates had a significantly higher digestibility than the control, however, 700 and 1000 g/t of Natuzyme also had significantly higher organic matter digestibility than 350 g/t of Natuzyme.

### 3.4. Bone Mineralization

The effects of Natuzyme super-dosing on tibia bone mineralization are presented in Table 8. The mineral content of the tibia bone, including calcium, phosphorous, magnesium, potassium, sodium, sulfur, iron, and zinc, were not altered by the multienzyme dose rates.

## 4. Discussion

### 4.1. Gut Morphology

The hypothesis of this trial was that multienzyme super-dosing would improve gut health via the breakdown of macromolecules, modulation of the broiler digestive physiology and manipulation of the microbial profile which would ultimately improve nutrient digestibility. The aim was to improve nutrient digestibility through enhancing the hydrolysis of macromolecules, improving gut development and increasing microbial diversity. This would enable a better surface area to volume ratio, thus a larger proportion of nutrients would be available and utilized by the bird, improving overall growth performance. The present experiment revealed no significant difference in villus height, crypt depth, VH/CD ratio, or goblet cell proliferation (Table 5) between any of the enzyme treatment groups. However, tendencies were observed that when the multienzyme dose rate increased, villi height and crypt depth increased in the duodenum and jejunum. Sharifi et al. [19] found that the addition of Natuzyme in corn–soybean diets significantly increased the villi height and improved nutrient digestibility. Ahmed et al. [20], Mazhari et al. [21], and Shakouri et al. [22] were in agreement, as they found that the inclusion of multienzymes significantly increased villus height and crypt depth, however, these were not super-dosed. However, Teirlynck et al. [23] demonstrated that including wheat in a ration evoked mucosal damage and villi fusion, which was typical of an inflammatory bowel condition. The standard diet formulation used in the present study contained approximately 40–45% wheat, which may have been a causative factor in the lack of significant difference between treatment groups and the presence of inflamed tissue (Figure 1).

The macroscopic gut samples did show observable differences between treatment groups regarding the presence of blood. Due to the variation, a scale (bloodiness scale) was created (Figure 1). Grade 4–5 necrotic tissue was recorded for a multitude of samples with the 1000 g/t enzyme level, however, in only a small number was grade 4–5 bloodiness observed for lower enzyme dose rates. It is hypothesized that the protease concentration in the 1000 g/t dose rate was too high for the bird to process, and accompanied by the endogenous protease excretion, may have begun digesting the gut wall. It was initially suspected that the presence of blood and necrotic tissue could also be due to a bacterial infection of the gut, however, upon completion of microbial profiling, it was disproved. This has not been recorded previously in the literature, so further investigation is required.

### 4.2. Microbial Community Diversity

Literature regarding super-dosing multienzyme effects on broiler intestinal microbiota is scarce, therefore, this study aimed to identify changes in the cecal microbial profiles influenced by multienzyme super-dosing, as improving nutrient availability will alter the abundance of specific bacteria as their food source has increased. The results of the present study highlight no differences in microbial diversity amongst all treatment groups, with no significant prevalence (*p* < 0.05) of any bacterial species being associated with a specific Natuzyme dose rate. These findings are supported by Lourenco et al. [24], who investigated the change in excreta microbial profiles in relation to protease and protein content in the diet. It was found that microbial richness was overall not changed in terms of observed OTUs and Chao1 index due to diet. Similar to the outcomes of the present trial, the Shannon index reported by Lourenco et al. [24] displayed no difference with enzyme inclusion, indicating a similar microbial profile across all treatment groups, in terms of species and richness.

As shown in Table 6, the relative abundance of bacteria was predominantly from the phylum Firmicutes, followed by Proteobacteria, Bacteroidetes, Actinobacteria, and other phyla. This trend has been also reported by Lourenco et al. [24] and Danzeisen et al. [25], with both finding Firmicutes as the predominant phylum in the chicken cecum content. Figure 5 reveals that of the 79 identified taxa, three were unique, 10 panned across several treatment groups, and the remaining 66 were core to all treatment groups. This indicates a relatively homogenous microbial pool across all treatment groups, which infers that altering multienzyme concentration alone does not influence microbial profile significantly. However, the lack of difference could also be due to the diet composition, which is found to have a large effect on microbial profile, by the modulation of physico-chemical properties and nutrient supply for specific microflora growth [22]. As all of the diets were formulated with approximately 40% wheat, 30% maize, and 20% soybean meal (Table 1), and although the dose rate of the multienzyme was increased (0 g/t, 350 g/t, 700 g/t, and 1000 g/t), the dietary composition was not changed, therefore, the same microbial consortia would proliferate in the same relative proportion in each treatment group.

There were three bacteria unique to a particular Natuzyme dose rate (Figure 5). Romboutsia spp. and Ruminococcus gauvreauii were isolated from 700 g/t Natuzyme. Ruminococcus has been reported to be positively correlated with good feed efficiency in broiler chickens, as this genus is known for its ability to degrade complex carbohydrates and fibers [26]. As work on these species has not been conducted in poultry, mapping of the human intestinal microbiome demonstrates that Romboutsia spp. is present in nutrient-rich environments where exogenous sources of amino acids and vitamins are abundantly available [27]. Whilst there is limited literature on this species functionality in poultry, the carbon sources it utilizes include D-arabinose, L-fucose, D-galactose, D-glucose, raffinose, and sucrose and it can produce acetate and formate [27]. This suggests a possible role in gut health promotion as it could modulate inflammation and oxidative stress [28,29]. The multienzyme nature of Natuzyme releases vitamins and minerals that are bound to compounds inaccessible by endogenous enzymes alone, such as protease, increasing protein digestibility, and phytase, increasing calcium, phosphorus, and zinc availability. Therefore, the presence of this species should not be surprising in super-dosed diets, however, this species did not appear in the D10 (1000 g/t Natuzyme) microbiome, but it remains unclear as to why. Further research is required on the role of Romboutsia spp. and Ruminococcus gauvreauii in the poultry digestive system. Similarly, Barnesiella spp. isolated from 1000 g/t Natuzyme has been reported to be one of the core cecal lumen bacteria and may contribute to hydrolyzing starch and other macromolecules, short-chain fatty acid formation, and feed conversion in broiler chicken [30]. Similarly, to Romboustia spp., the production of short chain fatty acids (SCFAs) could further promote gut health through the modulation of various immune-related host biological responses. Further studies are required on these species to ascertain their role in poultry digestive systems.

### 4.3. Nutrient Digestibility

The apparent ileal digestibility of nutrients is a direct indicator of bird health and gut functionality. In particular, the improvement of nutrient digestibility is commonly accompanied by an improvement in gut morphology, such as longer villi, which improves the surface area to volume ratio, thus enabling a wider surface for nutrients to absorb across. The results of the present study revealed significant improvement (*p* ≤ 0.001) in nutrient digestibility with increasing Natuzyme dose rates (Table 7).

As aforementioned, the primary enzymes in the formulation are phytase, xylanase, amylase, beta-glucanase, protease, and mannanase, thus it is a broad-spectrum application to feed, hence a significant increase in digestibility. The diets were approximately 40% wheat, 30% soybean meal, and 20% corn (Table 1) and combined, maize and wheat contain approximately 12% arabinoxylans, which are an antinutritional factor that increase intestinal viscosity which limits the absorption of nutrients in broiler chickens [7,31]. Incorporating xylanase and beta-glucanase into the diet enables a reduction in intestinal viscosity and eliminates the encapsulating effects of the non-starch polysaccharides in the gut and therefore a great overall improvement in nutrient digestibility. Soybean meal contains trypsin inhibitors, which block the degradation of protein, thus decreasing overall availability of protein in the diet [32], while corn and wheat contain phytic acid, which limits the bioavailability of phosphorus, calcium, zinc, and some amino acids [7]. Natuzyme contains protease, which alleviates the effect of trypsin inhibitors in soybean meal and improves protein hydrolysis in the presence of trypsin and therefore increases the digestible protein content of the diet [6]. Additionally, Natuzyme contains phytase, which breaks the bond between phytic acid, phosphorus, calcium, and zinc to increase mineral bioavailability, hence the significant improvement in nutrient digestibility in enzyme compared to no enzyme addition groups (Table 7) [33]. Similar results to those obtained in the present study are well recorded in the literature, with [33,34,35] demonstrating an improvement in overall digestibility when incorporating multienzymes into broiler diets.

### 4.4. Bone Mineralization

Bone mineral content and ash are good indicators of mineral utilization efficiency. It was expected that the multienzyme super-dosing may improve bone ash and mineral content. Our results, however, showed no differences in the tibia bone ash and mineral content when Natuzyme was included in the control diet at increasing dose rates. Our results are in line with the reports by Hajimohammadi et al. [36], who did not find significant differences in relative weight, length, density, and ash percentage of tibia bone when phytase was added to the negative control at 42 days of age. However, these outcomes are not in line with previous studies that have reported enhanced bone calcium and phosphorous content when phytase was supplemented in the negative control [37]. Similarly, Dilger et al. [38] and Francesch and Geraert [39] reported enhanced tibia ash and bone mineralization with phytase addition in the negative control diet. In our study, dietary calcium and phosphorous content was at the recommended level (positive control) and this might be a reason for the lack of response to multienzyme addition. The previous studies used negative control and positive control diets and the enzyme addition restored the bone mineral content of the birds in a similar way to the positive control [37,38,39].

## 5. Conclusions

This study aimed to ascertain whether super-dosing multienzymes would improve gut morphology, change the cecal microbial profile, and improve nutrient digestibility in broiler chickens. Results revealed that Natuzyme super-dosing tended to improve gut morphology, and significantly enhanced nutrient digestibility. The microbial profile was not significantly altered by increasing the Natuzyme dose rate, however, three bacterial species were unique to a higher level of Natuzyme in the diet. It was concluded that a dose of 700 g/t could be recommended to optimize gut morphology, nutrient digestibility, and support gut microbial diversity. Further investigations are required to study the functions and roles of Romboutsia spp., Ruminococcus gauvreauii, and Barnesiella spp. in the poultry digestive system and identify potential links between these species and improved feed utilization and efficiency.

## Figures and Tables

**Table 1:** Ingredient composition of the experimental diets ^1^.

Diet Composition (%)	Starter (1–14 d)	Grower (15–28 d)	Finisher (29–42 d)
Wheat	40.00	40.00	45.00
Corn	20.47	20.00	21.01
Soybean meal	29.83	29.36	23.81
Soybean oil	4.27	5.89	5.84
L-Lysine HCL ^2^	0.49	0.35	0.34
DL-Methionine	0.41	0.34	0.31
L-Threonine	0.26	0.18	0.16
Limestone	1.52	1.39	1.26
Mono-calcium phosphate	1.74	1.51	1.32
Sodium bicarbonate	0.32	0.25	0.25
Salt	0.15	0.20	0.20
Vitamin/Mineral premix	0.50	0.50	0.50
Coccidiostat	0.05	0.05	0.00

^1^ Natuzyme was added at 0, 350, 700, and 1000 g/ton dose rates, in each diet for the starter, grower, and finisher phases; ^2^ Lysine hydrochloric acid.

**Table 2:** Calculated and analyzed nutrient composition of experimental diets.

Diet Composition (%)	Starter (1–14 d)	Grower (15–28 d)	Finisher (29–42 d)
Calculated composition			
ME ^1^ (MJ/kg)	11.92	12.34	12.55
Crude protein (%)	21.00	20.50	18.50
SID ^2^ Lysine (g/kg)	12.80	11.50	10.20
SID Met + Cys (g/kg)	9.50	8.70	8.00
SID Threonine (g/kg)	8.60	7.70	6.80
Calcium (g/kg)	9.60	8.70	7.80
Avail. phosphorous (g/kg)	4.80	4.35	3.90
Sodium (g/kg)	1.60	1.60	1.60
Analyzed composition			
Dry matter (g/kg)	94.95	94.66	94.84
Crude protein (%)	20.03	18.20	19.59
Crude fat (g/kg)	61.11	77.00	76.44
Crude fiber (g/kg)	24.84	24.57	23.99
Calcium (g/kg)	11.90	10.40	8.00

^1^ Metabolizable energy. ^2^ Standardized ileal digestible.

**Table 3:** Enzyme activity levels in Natuzyme product.

Natuzyme Components	Enzyme Activity (u/g)
Xylanase	10,000
b-Glucanase	700
Phytase	1500
Alpha-amylase	400
Cellulase	6000
Protease	700
Mannanase	400

**Table 4:** Natuzyme expected vs. actual activity at different dose rates.

Natuzyme Dose Rate ( g/t)	Expected Activity (U/g)	Experimental Value (U/g)
350	1.27	1.20
700	2.54	2.51
1000	3.63	3.59

**Table 5:** The effects of Natuzyme dose rates on gut morphology of 42-day old broiler chickens.

Parameter	Experimental Diets				SEM	*p*-Value
	Control	350 g/t	700 g/t	1000 g/t		
Duodenum						
Villus height (μm)	956.32	995.36	1128.88	1204.45	98.57	0.06
Crypt depth (μm)	115.32	79.57	113.70	121.23	15.72	0.23
Villus width (μm)	1083.67	1084.41	1245.53	1132.29	125.57	0.78
VH/CD ^1^	9.12	13.02	10.63	11.14	1.78	0.50
Number of goblet cells	26.11	20.53	29.11	23.69	3.79	0.44
Jejunum						
Villus height (μm)	808.41	668.62	916.56	892.61	80.20	0.09
Crypt depth (μm)	92.79	92.18	146.19	103.87	16.42	0.10
Villus width (μm)	1121.64	827.72	804.57	1106.53	112.22	0.11
VH/CD	9.51	6.81	6.94	9.04	1.47	0.38
Number of goblet cells	17.94	18.03	14.06	15.78	3.87	0.87
Ileum						
Villus height (μm)	640.82	639.33	629.27	641.81	64.02	0.99
Crypt depth (μm)	111.61	107.93	116.65	118.98	25.65	0.99
Villus width (μm)	953.14	1063.70	1158.59	1192.81	147.04	0.10
VH/CD	6.62	6.73	6.63	6.38	1.17	0.99
Number of goblet cells	13.67	14.89	17.17	17.17	2.13	0.14

^1^ VH/CD = villus height to crypt depth ratio. Data are the means ± SEM of six replicates per treatment.

**Table 6:** Relative abundance of bacterial phyla (combined for all diets).

Phyla	Relative Abundance
Firmicutes	66.67%
Proteobacteria	7.58%
Bacteroidetes	6.06%
Actinobacteria	6.06%
Other phyla	<8%

**Table 7:** Nutrient apparent ileal digestibility of 42-day-old broiler chickens fed with Natuzyme super-dosed diets.

Parameter	Experimental Diets *				SEM	*p*-Value
	Control	350 g/t	700 g/t	1000 g/t		
Nitrogen digestibility %	51.0 ^a^	56.7 ^a^	70.5 ^b^	74.9 ^b^	2.4	<0.001
Organic matter %	55.2 ^a^	65.3 ^b^	74.6 ^c^	73.7 ^c^	0.5	<0.001

* Values with different superscripts a–c are statistically different (*p* < 0.05). Data are the means ± SEM of six replicates per treatment.

**Table 8:** Bone mineralization of 42-day-old broiler chickens fed with enzyme super-dosed diets.

Parameter, g/kg ash	Experimental Diets				SEM	*p*-Value
	Control	350 g/t	700 g/t	1000 g/t		
Total ash *	42.05	42.23	42.97	41.84	0.75	0.73
Calcium	374.16	373.42	376.14	373.35	1.94	0.42
Phosphorous	181.88	180.34	180.87	179.75	0.69	0.17
Magnesium	7.78	7.61	7.49	7.54	0.08	0.08
Potassium	4.89	4.78	4.54	4.58	0.43	0.64
Sodium	10.99	10.81	10.31	10.37	0.39	0.31
Sulfur	1.70	1.59	1.37	1.83	0.47	0.40
Iron	241.91	242.76	230.81	212.80	12.76	0.32
Zinc	267.55	271.38	275.46	264.82	6.48	0.68

* g/kg DM. Data are the means ± SEM of six replicates per treatment.

## Supplementary Materials

The following are available online at https://www.mdpi.com/2076-2615/11/1/1/s1, Figure S1: Rarefaction plot indicating total number of sequenced reads per sample and richness.
