# Supplementary material for: Multienzyme Super-Dosing in Broiler Chicken Diets: The Implications for Gut Morphology, Microbial Profile, Nutrient Digestibility, and Bone Mineralization
Source: Animals (Basel). 2020 Dec 22;11(1):1. doi: 10.3390/ani11010001 (PMC7821924; doi:10.3390/ani11010001)
Supplement: Supplementary file 1 [file animals-11-00001-s001.pdf]

Supplementary Materials: Multienzyme Super-Dosing in Broiler Chicken Diets: The Implications for Gut Morphology, Microbial Profile, Nutrient Digestibility, and Bone Mineralization

Jacoba Madigan-Stretton, Deirdre Mikkelsen and Elham Assadi Soumeh

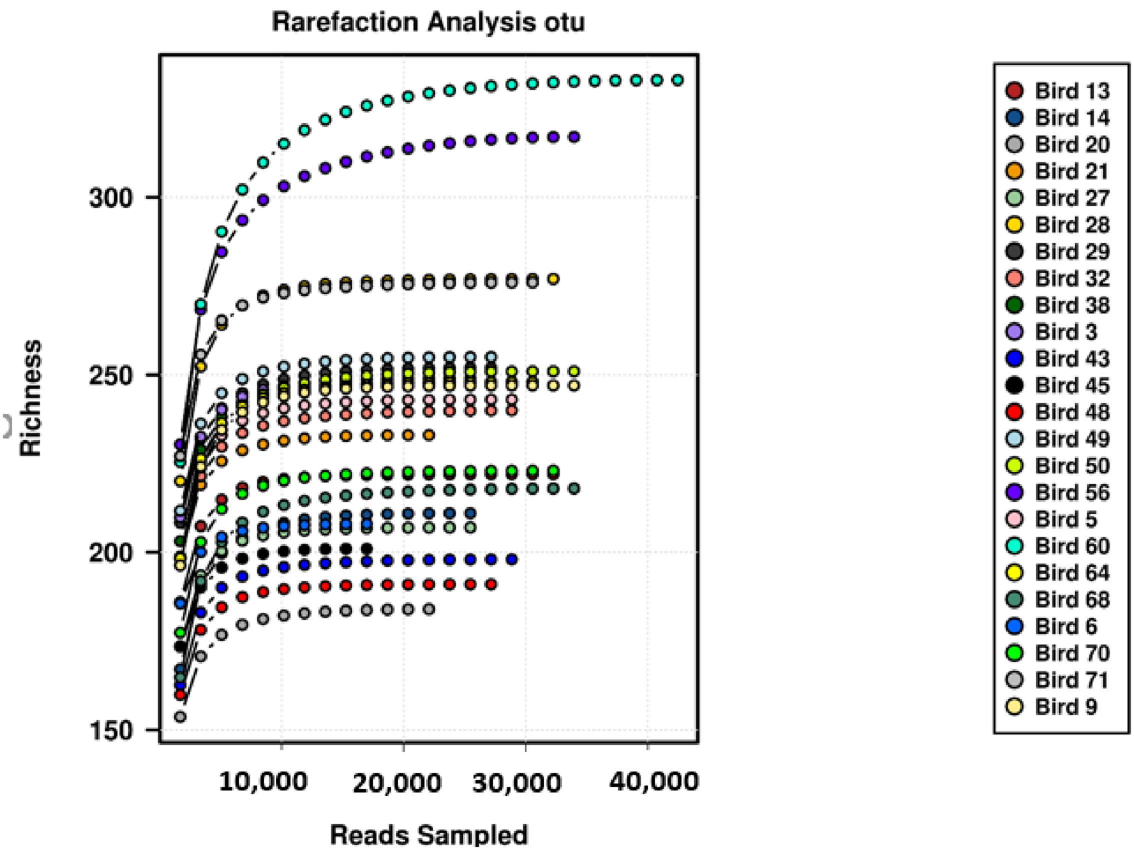

Supplementary Material Figure S1: Rarefaction plot indicating total number of sequenced reads per sample and richness, allowing for determining if appropriate sequence depth per sample was obtained, thereby assuring robust representation of microbial community diversity in each sample.
